# The Dynamic Mechanical Properties and Damage Constitutive Model of Ultra-High-Performance Steel-Fiber-Reinforced Concrete (UHPSFRC) at High Strain Rates

**DOI:** 10.3390/ma17030703

**Published:** 2024-02-01

**Authors:** Xiao Lv, Yan Li, Hui Guo, Wenbiao Liang, Yue Zhai, Le Li

**Affiliations:** 1 School of Geology Engineering and Geomatics, Chang’an University, Xi’an 710072, China; lvxiao135@163.com (X.L.); zy@chd.edu.cn (Y.Z.); lile@chd.edu.cn (L.L.); 2 School of Civil Engineering and Architecture, Southwest University of Science and Technology, Mianyang 621010, China; guohui56789@126.com; 3 Shock and Vibration of Engineering Materials and Structures Key Laboratory of Sichuan Province, Southwest University of Science and Technology, Mianyang 621010, China; 4 School of Sciences, Chang’an University, Xi’an 710072, China

**Keywords:** UHPSFRC, dynamic properties, damage constitutive model, high strain rate, steel fiber content, strength increase factor

## Abstract

A high strain rate occurs when the strain rate exceeds 100 s^−1^. The mechanical behavior of materials at a high strain rate is different from that at middle and low strain rates. In order to study the dynamic compressive mechanical properties of ultra-high-performance steel-fiber-reinforced concrete (UHPSFRC) at high strain rates, an electro-hydraulic servo universal testing machine and a separate Hopkinson pressure bar (SHPB) with a diameter of 120 mm were used, respectively. A quasi-static compression test (strain rate 0.001 s^−1^) and impact compression test with a strain rate range of 90~200 s^−1^ were carried out to study the failure process, failure mode, and stress–strain curve characteristics of UHPSFRC at different strain rates and quantify the strain rate strengthening effect and fiber toughening effect. Based on the statistical damage theory and energy conversion principle, a dynamic damage constitutive model considering the effects of strain rate and fiber content was constructed. The results showed that the rate correlation of UHPSFRC and the fiber toughening properties showed a certain coupling competition mechanism. When the fiber content was less than 1.5%, with an increase in the steel fiber content, the crack initiation and propagation time of the specimen was extended, and the strain rate sensitivity gradually decreased. When the fiber content was 2%, the impact compressive strength of the specimen was optimal. Compared with UHPC, the dynamic increase factor (DIF) of UHPSFRC was significantly lower. The dynamic damage constitutive model established in this paper, considering the influence of strain rate and fiber content, has a good applicability and can describe the mechanical behavior of UHPSFRC at a high strain rate.

## 1. Introduction

A high strain rate is defined when the strain rate exceeds 100 s^−1^ [1]. With the frequent occurrence of natural disasters and the increase in social instability factors, the problems of high strain rate failure such as vibration, shock, and explosion in the whole life of concrete engineering are becoming more and more prominent [2,3]. Ultra-high-performance concrete (UHPC) is widely regarded as an excellent protective material due to its superior strength [4] and strong impact resistance [5], and the study of its mechanical behavior at high strain rates can provide certain theoretical references for explosion-proof and impact resistance engineering design.

At present, the dynamic mechanical response of UHPC is the focus of limited existing research. Relevant studies have shown that an SHPB device is an effective testing means for testing the dynamic mechanical properties of concrete materials in the range of 10~1000 s^−1^ strain rate [6]. For example, for UHPC, some scholars have used SHPB devices to study its hardening effect at a strain rate of 2 s^−1^ [7] and the influence law of fiber content and matrix strength on dynamic mechanical properties at a strain rate from 10^−6^ to 5 s^−1^ [1]. Some other scholars have explored the mechanical properties of UHPC such as compressive strength, tensile strength, and elastic modulus under higher strain rate conditions (30 s^−1^) [8] and a wider strain rate range (0–80 s^−1^) [9]. The results have shown that UHPC exhibits different mechanical behaviors at different strain rate intervals, that is, the mechanical properties of the material have obvious strain rate classifications.

As a typical representative of UHPC, UHPSFRC is a new generation of concrete with a higher performance and strength than traditional concrete. Despite its great application potential, there is still limited technical information about this material, including its mechanical behavior and constitutive models at high strain rates. Although recognizing that the strain rate corresponding to the blast impact load faced by concrete engineering during its service exceeds 100 s^−1^, some scholars have carried out a series of dynamic compression tests of ultra-high-performance fiber-reinforced concrete at a high strain rate [10,11,12], providing very valuable test data and research conclusions for the research in this field. However, there is little research information on UHPSFRC, especially on the constitutive model that considers both strain rate effect and fiber content.

The constitutive model of UHPSFRC is crucial for analyzing the safety and stability of corresponding concrete engineering structures [13]. At present, constitutive models of concrete materials are mainly divided into two categories: elastic–plastic hydraulic models and elastic–plastic damage models [14]. The elastic–plastic hydraulic model is characterized by the decoupling of the model stress deviation from the spherical tensor and the combination of the equation of state (EOS) to capture nonlinear volumetric compaction, making it suitable for describing the local response of materials under high hydrostatic pressure loading. For example, for high-speed projectile penetration or a close-in explosion scenario, typical elastic–plastic water-code concrete models, namely the HJC, RHT, and KCC models, were initially proposed [15,16,17]. The elastic–plastic hydraulic model can reflect the influence of fibers on the adjustment of the yield surface and strain rate of the HJC model [15] and can also calibrate the failure surface and dynamic increase factor [17]. The elastic–plastic damage model can fully reflect the sensitivity of the fracture energy, plastic volume change, and other parameters [18]. Both the elastic–plastic hydraulic model and the elastic–plastic damage model are established by adjusting the parameter values of the traditional concrete constitutive model, which is a traditional and classical constitutive model construction method. For UHPSFRC, a constitutive model considering the coupling effects of strain rate and fiber content is very important for fully reflecting the actual mechanical behavior of UHPSFRC. Although some studies have proposed a constitutive model based on the two-parameter Weibull function, which can be used to evaluate the effects of fiber orientation and strain rate [19], its applicable scope is still limited in scenarios where fiber content plays a key role.

In order to study the dynamic compression mechanical properties of UHPSFRC at high strain rates, quasi-static compression tests (strain rate 0.001 s^−1^) and impact compression tests with strain rate ranging from 90 to 200 s^−1^ were carried out using an electro-hydraulic servo universal testing machine and separate Hopkinson pressure bar (SHPB) device with diameter of 120 mm, respectively. The failure process, failure mode, and stress–strain curve characteristics of UHPSFRC at different strain rates were studied, and the effect of strain rate strengthening and fiber toughening were quantified. Based on the statistical damage theory and the energy conversion principle, a dynamic damage constitutive model considering the influence of strain rate and fiber content was constructed, which may provide a theoretical reference for the lifetime design and safe service of UHPSFRC.

## 2. Experiments

### 2.1. Raw Material

The raw materials of UHPSFRC are provided by North Branch of China Construction Second Engineering Bureau Co., Ltd. (Shenyang, China), which are shown in Table 1. The cement is Portland cement P·O 42.5. The steel fiber types are high-strength, round-section straight steel fibers with diameters ranging from 0.18 to 0.22 mm and length-to-diameter ratios from 65 to 100. The volume content of the steel fiber in UHPSFRC is set to 0%, 1%, 1.5%, and 2%, respectively.

### 2.2. Preparation of UHPSFRC

**Figure 1:**
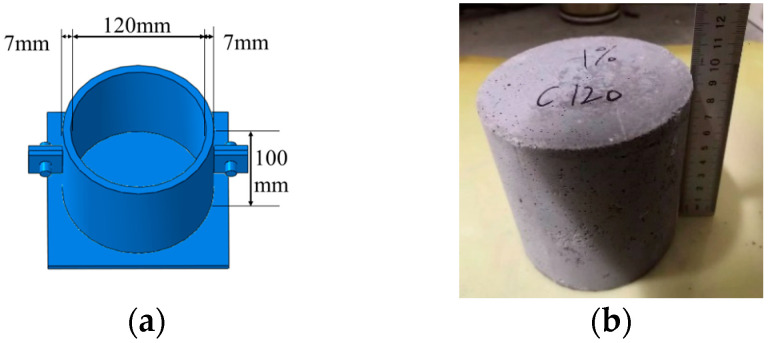
The mold design diagram and specimen of UHPSFRC. (**a**) Mold design diagram and (**b**) specimen.

The preparation process of the specimen was as follows: firstly, raw materials and water were weighed separately and poured into a mixer. After stirring for 3~4 min, the steel fibers were slowly added while continuously stirring, and then vibrated while stirring, so that the steel fiber distributed evenly. According to the ASTM C1856/C1856M-17 standard [20], the specimens were first cured at 25 °C for 24 h, then steam-cured at a temperature above 80 °C for 48 h, and finally cured at 20 ± 2 °C for at least 28 days. The mold design diagram and UHPSFRC specimen are shown in Figure 1. To ensure consistent stress distribution, the concrete molds were all cylindrical, with a diameter of 120 mm and a height of 100 mm.

### 2.3. Static and Dynamic Compression Experiments

According to the Chinese standard GB/T 50081-2019 [21], quasi-static compression tests were carried out on an electro-hydraulic servo universal test machine (Shock and Vibration of Engineering Materials and Structures Key Laboratory of Sichuan Province, Southwest University of Science and Technology, Mianyang, China) with a loading rate of 0.9 MPa/s, which can provide important basic comparative data for the study of UHPSFRC at different strain rates.

**Figure 2:**
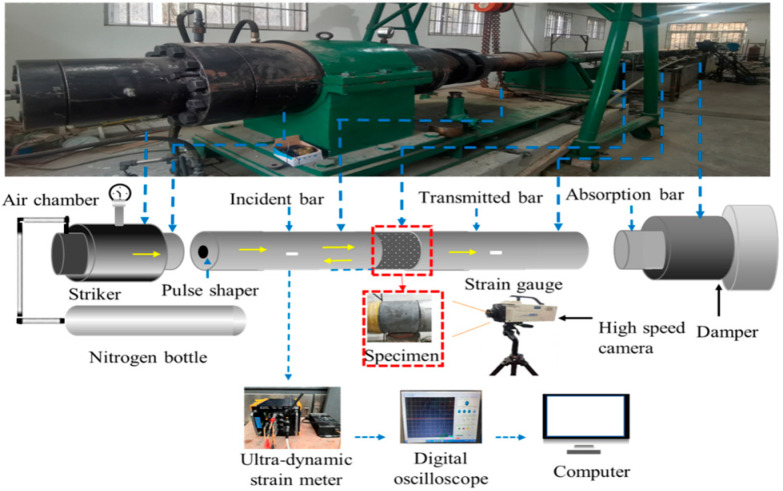
The diagram of the SHPB test device.

In order to achieve the high strain rate of more than 100 s^−1^ matched with the impact loading, a series of dynamic uniaxial compression tests were conducted on an SHPB device with a large diameter of 120 mm (Shock and Vibration of Engineering Materials and Structures Key Laboratory of Sichuan Province, Southwest University of Science and Technology, Mianyang, China), as shown in Figure 2. The impact pressures were set at 3.4 MPa, 3.8 MPa, 4.2 MPa, 4.6 MPa, and 5 MPa, corresponding to strain rates of 90 s^−1^, 120 s^−1^, 140 s^−1^, 170 s^−1^, and 200 s^−1^, respectively.

**Figure 3:**
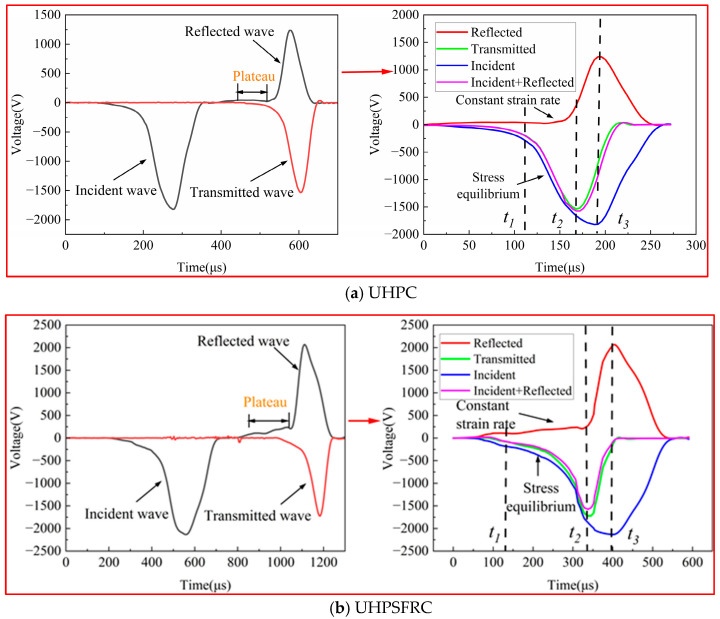
Time history curves of voltage for UHPC (**a**) and UHPSFRC (**b**).

To improve the accuracy of the experiment, the “three-wave method” [22] was used to analyze the dynamic test data, and the waveform time history curves of UHPC and UHPSFRC are shown in Figure 3. 

As depicted in Figure 3, the specimen started to satisfy the constant strain rate loading from the moment when the reflected wave entered the platform phase at time t_1_ [23]. The time interval from t_2_ to t_3_ refers to the time elapsed from the peak of the transmitted wave to the peak of the incident wave, and also represents the time for the specimen from reaching the stress peak until complete failure. The duration for UHPSFRC from t_2_ to t_3_ was 72 s, while for UHPC it was 28 s. Compared to UHPC, UHPSFRC has a longer buffer time from initial damage to complete failure and demonstrates a stronger impact resistance. 

## 3. Results and Discussion

### 3.1. Results of Quasistatic Compression Tests

**Figure 4:**
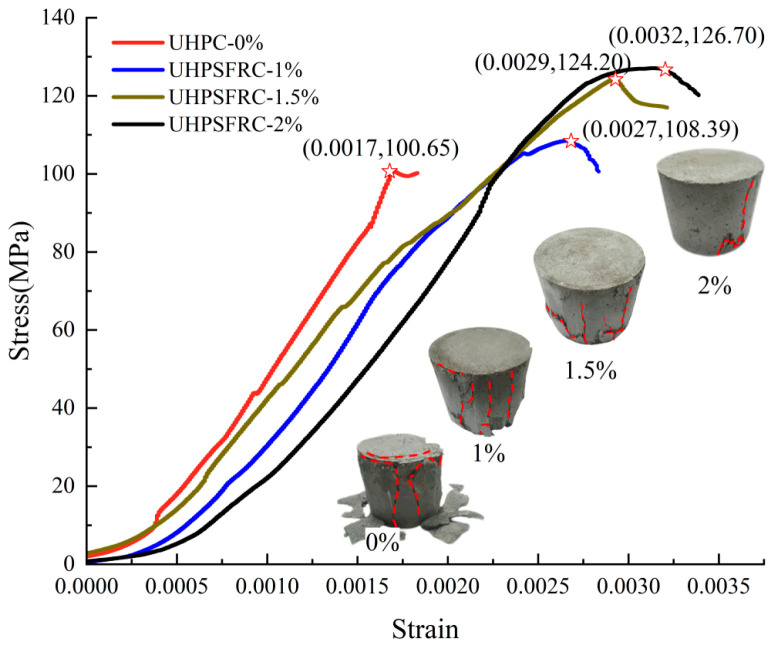
Stress–strain curves and failure modes of UHPSFRC under quasi-static loads.

The stress–strain curves and failure modes of UHPSFRC with four fiber contents (0%, 1%, 1.5%, and 2%) under quasi-static compression loads are presented in Figure 4.

It can be seen from Figure 4 that the curves of UHPSFRC are relatively slow in the elastic stage, and the elastic modulus of UHPSFRC is slightly lower than that of UHPC. Meanwhile, with an increase in the fiber content, the peak strain and peak stress of UHPC and UHPSFRC increase significantly. The main failure characteristic of UHPC specimens is quasi-brittle shear failure, with cracks propagating from top to bottom and ultimately extending throughout the specimen. In contrast, UHPSFRC specimens exhibit brittle–ductile failure characteristics, with the formation of tangential tension and cracks on the lateral surface during the static compression process. Due to the bonding effect of steel fibers, the tensile stress near the crack is effectively shared and the expansion of internal cracks is effectively inhibited. Especially when the fiber content is 2%, the spalling degree for UHPSFRC is relatively mild and the quasi-static compressive strength is the highest.

### 3.2. Dynamic Failure Prosess

**Figure 5:**
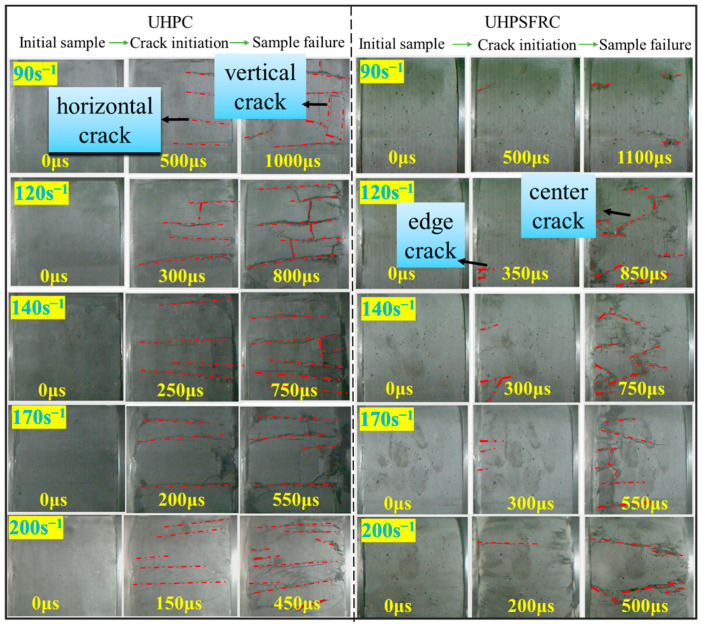
Failure processes of UHPC and UHPSFRC at different strain rates.

In order to fully restore the failure process of the specimen, a high-speed camera system (MEMRECAMHX-4E, Mianyang, China) was utilized to visualize the dynamic microdamage of the UHPSFRC and UHPC samples. The camera was set to automatic trigger mode at a sampling frame rate of 20,000 frames per second. The distinct failure processes at different strain rates (90, 120, 140, 170, and 200 s^−1^) are shown in Figure 5.

It can be seen from the microscopic images in Figure 5 that the damage degree of UHPSFRC was significantly lower than that of UHPC. The initial cracks in the UHPC specimens were longitudinal cracks, and the distribution and number of newly formed cracks and derived fractures increased with an increase in the strain rate. The horizontal cracks gradually spread and extended into vertical cracks at a 90° angle from the direction of the original cracks, eventually splitting the specimen into multiple smaller prisms. The crack initiation in the UHPSFRC specimens was from both ends towards the center, and the crack propagation speed slowed down due to the addition of the steel fibers. When the strain rate was from 90 to 170 s^−1^, the internal cracks inside the specimen were short and fine; however, when the strain rate increased to 170 s^−1^, the internal cracks in the specimens started to expand obviously, but the samples were not completely broken. When the strain rate exceeded 170 s^−1^, the damage degree of the specimen was obviously deepened.

**Figure 6:**
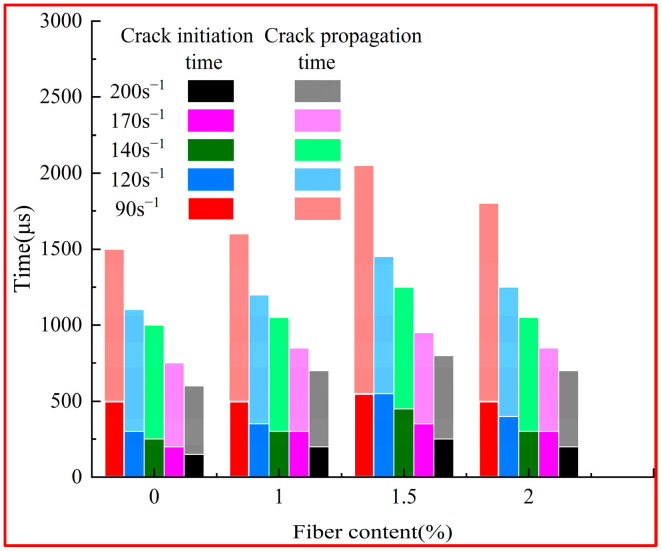
Time-lapse diagram of crack propagation in UHPSFRC with different fiber contents.

The initiation and the propagation time for cracks in UHPC and UHPSFRC were measured under impact loads, and the time-lapse diagram of the crack evolution process is shown in Figure 6.

According to Figure 6, the crack initiation and propagation time both declined with an increase in the strain rate. Because of the addition of steel fibers, the initiation and propagation time of cracks in the UHPSFRC specimens were postponed. For instance, when the strain rate was 120 s^−1^, the crack initiation and propagation time for UHPC were 300 μs and 800 μs, while at fiber contents of 1%, 1.5%, and 2%, the crack initiation time for UHPSFRC was extended by 6.3%, 12.5%, and 6.3%, and the crack propagation time for this material was extended by 10%, 50%, and 30%, respectively. When the fiber content was 1.5%, the crack propagation rate was significantly reduced.

### 3.3. Results of Dynamic Compression Tests 

**Figure 7:**
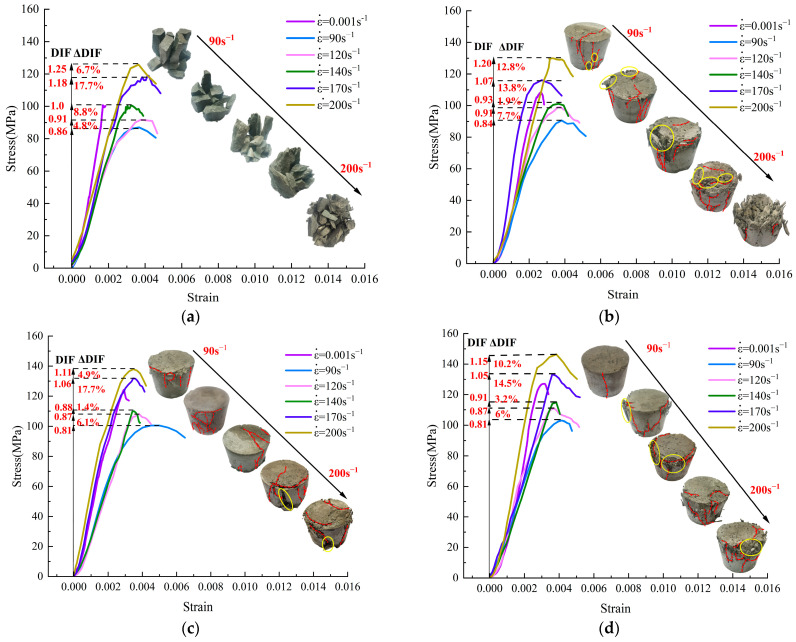
Failure modes and stress–strain curves. (**a**) UHPC-0%; (**b**) UHPSFRC-1%; (**c**) UHPSFRC-1.5%; and (**d**) UHPSFRC-2%.

The stress–strain curves and failure modes of UHPC and UHPSFRC at five strain rates (90, 120, 140, 170, and 200 s^−1^) are shown in Figure 7. To quantify the strain rate dependence of dynamic strength, the value of DIF [24] and increment of DIF (ΔDIF) were calculated, which are defined as follows:

(1) $$\mathrm{DIF}=\frac{\sigma_{d}}{\sigma_{s}}$$

(2) $$\Delta \mathrm{DIF}=\frac{\left(\sigma_{d,n+1}-\sigma_{d,n}\right)}{\sigma_{s}}$$

where σ_d_ and σ_s_ are the dynamic and quasi-static strength, respectively, and σ_d,n+1,_ and σ_d,n_ are the strength values corresponding to the adjacent strain rate.

In Figure 7, the distributions of cracks are indicated by red lines and the area of aggregate spalling is marked with yellow circles. With an increase in the strain rate, the density and width of cracks in the sample gradually increased, accompanied by the spalling of fine aggregate. The damage level of UHPSFRC was significantly lower than that of UHPC. The fracture orientation of UHPC was aligned with the direction of principal stress, and most of the fragments were prismatic in shape. However, the damage mode of UHPSFRC was cracking rather than fragmentation, and the damage degree decreased gradually with an increase in the fiber content. When the fiber content was 1%, the impact end faces of the specimen exhibited a rough and uneven surface texture. In contrast, the specimens with 1.5% and 2% fiber contents were more intact after impact loads, the end faces of the specimens were relatively smooth, and tensile cracks appearing in the weak areas of the lateral interface were limited. Due to the random distribution of steel fibers, a three-dimensional framework was formed inside the specimen and the expansion of cracks was effectively prevented.

The peak stress was improved as the strain rate rose from 90 s^−1^ to 200 s^−1^ and the dynamic peak strain was larger than the static one. In Figure 7b, the static peak strain value for UHPSFRC-1% is 0.0026, whereas the dynamic peak strain values range from 0.0034 to 0.0039, indicating a stronger deformation resistance capability under impact dynamic loads. The maximum dynamic peak stress of UHPSFRC was obtained when the fiber content was 2%. After the peak stress is reached, the stress curve shows an irregular downward trend and there is no obvious residual deformation.

**Figure 8:**
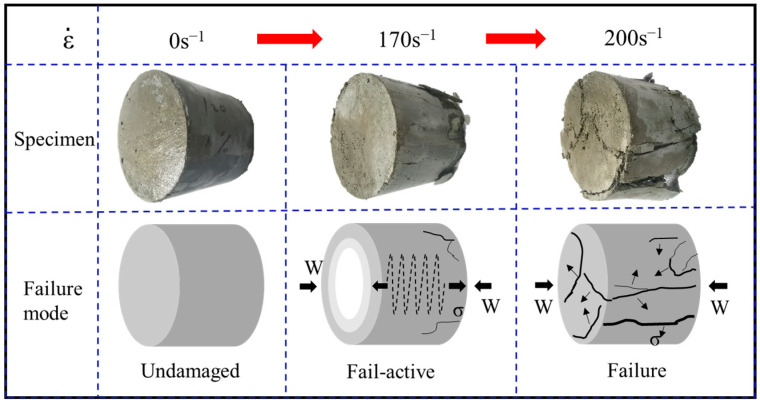
Failure mode diagram.

With an increase in the strain rate, the DIF values of UHPC and UHPSFRC both exhibited a gradual upward trend, as shown in Figure 7a–d. When the dynamic strain rate ranged from 90 s^−1^ to 170 s^−1^, the increase in ΔDIF was relatively small and the dynamic peak stress value for UHPSFRC was lower than the quasi-static one. When the strain rate exceeded 170 s^−1^, the ΔDIF value was significantly improved. There is, perhaps, one main conclusion that can be drawn, that is, 170 s^−1^ is a strain rate threshold for a significant change in strain rate sensitivity for UHPSFRC. When the strain rate is lower than 170 s^−1^, the internal elastoplastic properties of the specimen do not fail, but when the strain rates exceed this threshold, a portion of energy is allocated to inhibiting the generation and expansion of cracks, and the remaining energy is used to enhance stress, resulting in an increase in the ΔDIF value. The corresponding failure modes are depicted in Figure 8. 

## 4. Statistical Damage Constitutive Model

### 4.1. DIF Constitutive Models

Currently, many empirical models have been proposed to describe the correlation between the DIF and strain rate of concrete-like materials. For a comparative analysis, some commonly used models have been selected here:

(1) Exponential function.

Based on the dynamic tests for concrete-like materials at strain rates from 1~1000 s^−1^, a DIF model was obtained by Lu and Xu et al. [25]. The equations are:

(3) $$\mathrm{DIF}=1+0.15{\overset{\cdot }{\varepsilon }}^{0.2}+0.0013{\overset{\cdot }{\varepsilon }}^{0.2}$$

(2) Power function.

A series of dynamic compression tests were tested on solid and annular cylindrical cement mortar specimens with different aspect ratios, respectively, by Al-Salloum et al. [26], and a DIF model given by Equation (4) was proposed based on the test data:

(4) $$\mathrm{DIF}=(3.54\overset{\cdot }{\varepsilon }+430.6)/(\overset{\cdot }{\varepsilon }+447.3)$$

(3) Logarithmic function.

Based on the ADINA finite element analysis for concrete, a DIF rate-dependent constitutive model was proposed by Tedesco and Ross [27]. The specific equation is as follows:

(5) $$\left\{\begin{array}{l} \mathrm{DIF}=0.00965\log\overset{\cdot }{\varepsilon }+1.058\geq 1.0,\overset{\cdot }{\varepsilon }\leq 63.1s^{-1}\\ \mathrm{DIF}=0.758\log\overset{\cdot }{\varepsilon }-0.289\leq 2.5,\overset{\cdot }{\varepsilon }\geq 63.1s^{-1} \end{array}\right.$$

A DIF equation applicable to the strain rate range from 250 to 1700 s^−1^ was proposed by Grote et al. [28]. The equations are:

(6) $$\left\{\begin{array}{l} \mathrm{DIF}=0.0235\log\overset{\cdot }{\varepsilon }+1.17,\overset{\cdot }{\varepsilon }\leq 266s^{-1}\\ \mathrm{DIF}=0.882{\left(\log\overset{\cdot }{\varepsilon }\right)}^{3}-4.4{\left(\log\overset{\cdot }{\varepsilon }\right)}^{2}-2.64,\overset{\cdot }{\varepsilon }>266s^{-1} \end{array}\right.$$

With reference to the models proposed by Tedesco and Ross [27] and Grote et al. [28], a DIF model considering the lateral inertial constraints for concrete-like materials was developed by Li and Meng [29], as shown in Equation (7).

(7) $$\left\{\begin{array}{l} \mathrm{DIF}=0.03438(\log\overset{\cdot }{\varepsilon }+3)+1,\overset{\cdot }{\varepsilon }\leq 100s^{-1}\\ \mathrm{DIF}=1.729{\left(\log\overset{\cdot }{\varepsilon }\right)}^{2}-7.1372\log\overset{\cdot }{\varepsilon }+8.5303,\overset{\cdot }{\varepsilon }>100s^{-1} \end{array}\right.$$

Zhou and Hao obtained a DIF equation with a critical strain rate of 10 s^−1^ through numerical simulation [30]. The equations are:

(8) $$\left\{\begin{array}{l} \mathrm{DIF}=0.0225\log\overset{\cdot }{\varepsilon }+1.12,\overset{\cdot }{\varepsilon }\leq 10s^{-1}\\ \mathrm{DIF}=0.2713{\left(\log\overset{\cdot }{\varepsilon }\right)}^{2}-0.3563\log\overset{\cdot }{\varepsilon }+1.2275,\overset{\cdot }{\varepsilon }>10s^{-1} \end{array}\right.$$

**Figure 9:**
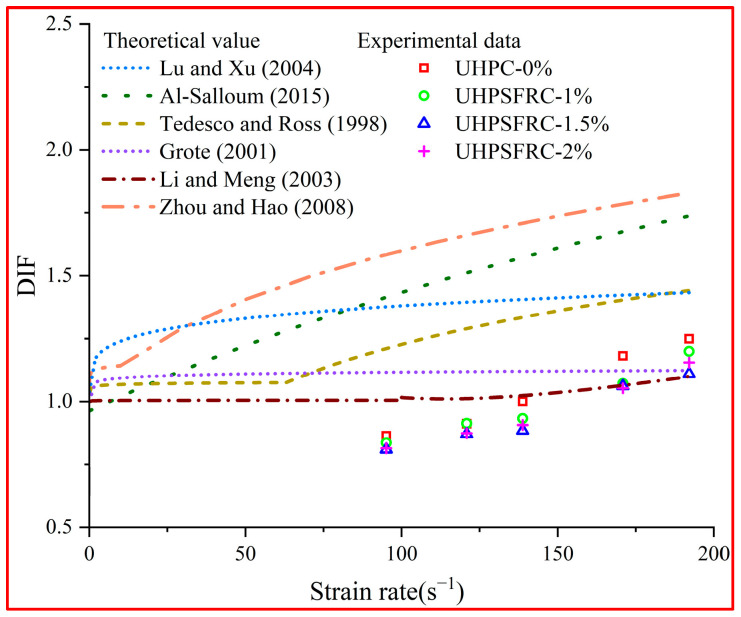
Comparison of DIF model [25,26,27,28,29,30].

In this study, the theoretical values of the above experience model were compared with the experimental data, as shown in Figure 9. 

**Figure 10:**
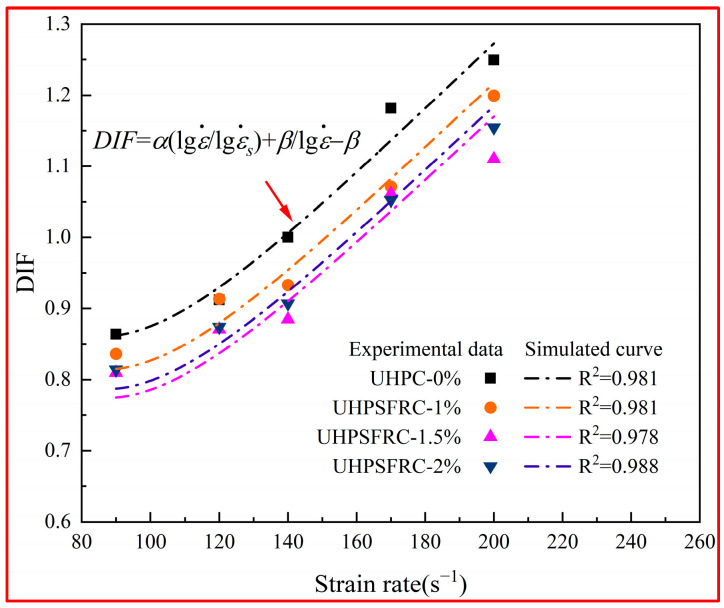
Fitting model of DIF.

As can be seen from Figure 9, the DIF curves exhibit an upward trend with an increase in the strain rate. The DIF values given by Li and Meng [29] and Grote [28] are closer to the experimental results in the strain rate range of 90~200 s^−1^, while the values of other empirical models are slightly higher than the measurements. This phenomenon may be due to the influence of steel fibers on UHPSFRC and the discretization of the model. Based on the model of Li and Meng [29] and Grote et al. [28], a new DIF-$\log\dot{\varepsilon }$ model for UHPSFRC was obtained by using MATLAB (R2022b), as presented in Equation (9) and Figure 10.

(9) $$\mathrm{DIF}=\alpha (\frac{\mathrm{lg}\dot{\varepsilon }}{\mathrm{lg}{\dot{\varepsilon }}_{s}})+\frac{\beta }{\mathrm{lg}\dot{\varepsilon }}-\beta$$

where $\dot{\varepsilon }$ is the strain rate, α is the strain rate sensitivity coefficient, β = 0.195${\dot{\varepsilon }}_{cr}$, and ${\dot{\varepsilon }}_{cr}$ is the strain rate when DIF = 1.

The goodness of fitting R^2^ values for the fitting curve are all greater than 0.97. It can be seen that the reported DIF data can be well predicted by the new DIF-log$\dot{\varepsilon }$ model. The specific parameters of the new DIF formula are shown in Table 2. 

The fitting results indicate that, at the same strain rate, the DIF value and strain rate sensitivity coefficient α of UHPC are higher than those of UHPSFRC. For instance, at 120 s^−1^, the DIF value of UHPC is 0.912 and the α value is 0.0719, whereas, for UHPSFRC at a fiber content of 1%, the corresponding DIF values are 0.913, 0.871, and 0.874, and the α values are 0.07166, 0.07146, and 0.07152, respectively. Due to the bridging ability of steel fibers to cracks, the strain rate sensitivity of UHPSFRC is low, the energy absorption toughness is enhanced, and the brittleness characteristics are mitigated. Compared with other fiber contents, the value of DIF and coefficient α at the 1.5% content are smallest and the buffering performance is the best, which is also confirmed in Section 3.2.

### 4.2. Establishment of the Statistical Damage Constitutive Model for UHPSFRC

**Figure 11:**
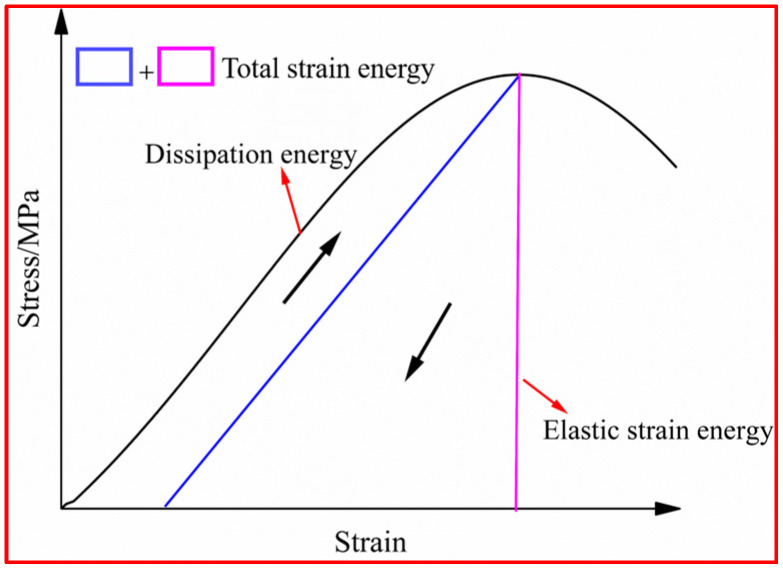
Energy conversion relationship in loading process.

During the dynamic compression process, the energy absorbed by the UHPSFRC specimen is gradually transformed into elastic strain energy, dissipative energy, electromagnetic energy, and radiation energy. However, electromagnetic energy and radiation energy have little effect under actual action. Therefore, the energy within the specimen is predominantly present in the form of dissipative energy and elastic strain energy [31], as shown in Figure 11. 

After reaching the peak stress at high strain rates, the specimen rapidly failed due to the inherent brittleness of concrete; thus, the constant strain rate loading is difficult to satisfy. Therefore, the mechanical property analysis for UHPSFRC primarily focuses on the stress–strain curve to the left of the peak stress [31]. The energy absorbed by the specimen under impact loading can be calculated as follows:

(10) $$W=W_{e}+W_{d}=\int_{0}^{\varepsilon }\sigma d\varepsilon$$

where *W_e_* is the elastic strain energy, *W_d_* is the dissipative energy of damage development, σ is the axial stress of the specimen, MPa, and ε is the axial strain.

Due to the inevitable existence of defects such as microcracks in UHPSFRC specimens, the internal damage is random under impact loads. The Weibull model [32] is a commonly used prediction model for evaluating the damage of concrete-like materials, which assumes that the damage probability density function of the dissipated energy elements in UHPSFRC follows the Weibull distribution, and the expression is as follows:

(11) $$\rho (\varepsilon )=\frac{r}{a}{\left(\frac{\varepsilon -u}{a}\right)}^{r-1}e^{\left[-{\left(\frac{\varepsilon -u}{a}\right)}^{r}\right]}$$

where *u* is the location parameter, *a* is the scale factor, and *r* is the shape parameter.

The specimen of UHPSFRC can be modeled as a system composed of numerous volumetric elements. With an increase in energy dissipation, the volume of damaged elements in UHPSFRC can be calculated as follows:

(12) $$V*=\int_{0}^{\varepsilon }V\rho (\varepsilon )d\varepsilon =\int_{0}^{\varepsilon }V\frac{r}{a}{\left(\frac{\varepsilon -u}{a}\right)}^{r-1}e^{\left[-{\left(\frac{\varepsilon -u}{a}\right)}^{r}\right]}d\varepsilon =V\left[1-e^{-{\left(\frac{\varepsilon -u}{a}\right)}^{r}}\right]$$

where V_0_ is the total volume elements and V* is the failed volume elements.

The dissipated energy can be integrated as follows:

(13) $$W_{d}=\int_{0}^{\varepsilon }(1-\frac{V*}{V})\sigma d\varepsilon =\int_{0}^{\varepsilon }(e^{-{\left(\frac{\varepsilon -u}{a}\right)}^{r}})\sigma d\varepsilon$$

According to the principle of strain coordination, the strain in the damaged portion in the UHPSFRC specimen is synchronized with the strain in the undamaged portion, which ensures consistency of the overall strain. Assuming the undamaged portion in the UHPSFRC specimen still satisfies the linear elastic deformation law, the elastic strain energy can be given below:

(14) $$W_{e}=\int_{0}^{\varepsilon }f(\dot{\varepsilon },\lambda )\varepsilon d\varepsilon$$

where $f(\dot{\varepsilon },\lambda )$ is the material parameters related to the strain rate and fiber content.

Based on energy dissipation theory [31] and according to Equations (10)–(14), a dynamic damage constitutive model for UHPSFRC can be derived:

(15) $$\sigma =\frac{\partial W}{\partial \varepsilon }=\frac{\partial W_{d}+\partial W_{e}}{\partial \varepsilon }=\frac{\partial \int_{0}^{\varepsilon }(e^{-{\left(\frac{\varepsilon -u}{a}\right)}^{r}})\sigma d\varepsilon +}{d\varepsilon }=\frac{r}{a}{\left(\frac{\varepsilon -u}{a}\right)}^{r-1}e^{\left[-{\left(\frac{\varepsilon -u}{a}\right)}^{r}\right]}+f(\dot{\varepsilon },\lambda )\varepsilon$$

**Figure 12:**
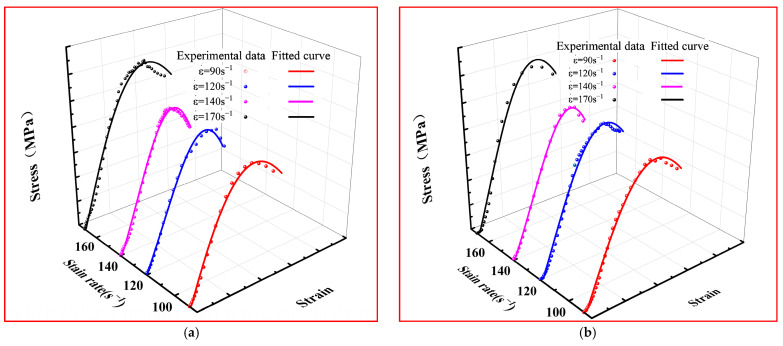

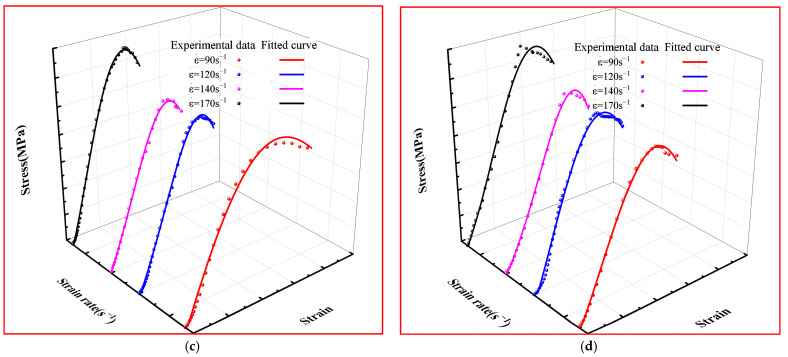
Experimental data and fitted curves of SHPB tests in the strain rate range of 90~170 s^−1^. (**a**) UHPC-0%; (**b**) UHPSFRC-1%; (**c**) UHPSFRC-1.5%; and (**d**) UHPSFRC-2%.

The fitting parameters *f(*$\dot{\varepsilon }$*,λ)*, *a*, *r*, and *u* in the energy damage constitutive model in the case of strain rates of 90, 110, 140, and 170 s^−1^ can be derived, which are presented in Figure 12 and detailed in Table 3.

As depicted in Figure 12 and Table 3, the fitting curve of the damage constitutive equation (Equation (12)) is in good agreement with the experimental data, and the goodness of fit R^2^ value is more than 0.95, It can be obtained that the stress–strain curves of UHPSFRC can be well described by the constitutive damage model in the strain rate range of 90~170 s^−1^.

**Figure 13:**
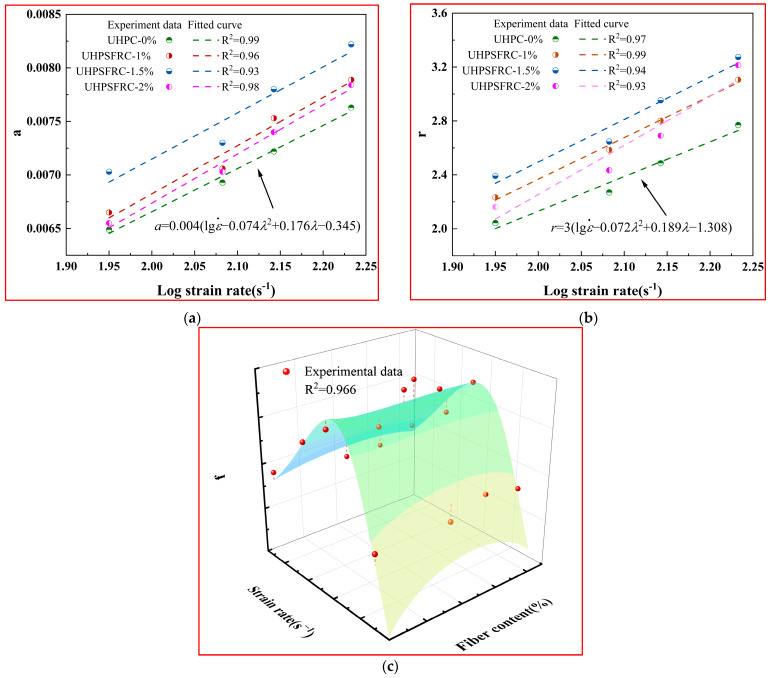
Variations of model parameters under different fiber contents and strain rates. (**a**) Regression analysis curve of parameter *a*; (**b**) regression analysis of parameter *r*; and (**c**) regression analysis diagram of parameter *f*.

The expressions for *f(*$\dot{\varepsilon }$*,λ)*, *a* and *r* can be derived through Equations (16)–(18). The relevant expressions of the parameters are illustrated in Figure 13.

(16) $$a=0.004(\mathrm{lg}\overset{\cdot }{\varepsilon }-0.074\lambda^{2}+0.176\lambda -0.345)$$

(17) $$r=3(\mathrm{lg}\overset{\cdot }{\varepsilon }-0.072\lambda^{2}+0.189\lambda -1.308)$$

(18) $$f(\dot{\varepsilon },\lambda )=\frac{22.3-0.11\dot{\varepsilon }+5.14\lambda +-2.06\lambda^{2}-0.11\lambda^{3}}{1-0.007\dot{\varepsilon }+0.1\lambda -0.04\lambda^{2}}$$

The variation rules of *f(*$\dot{\varepsilon }$*,λ)*, *a*, and *r* are shown in Figure 13. 

### 4.3. Model Validation

**Figure 14:**
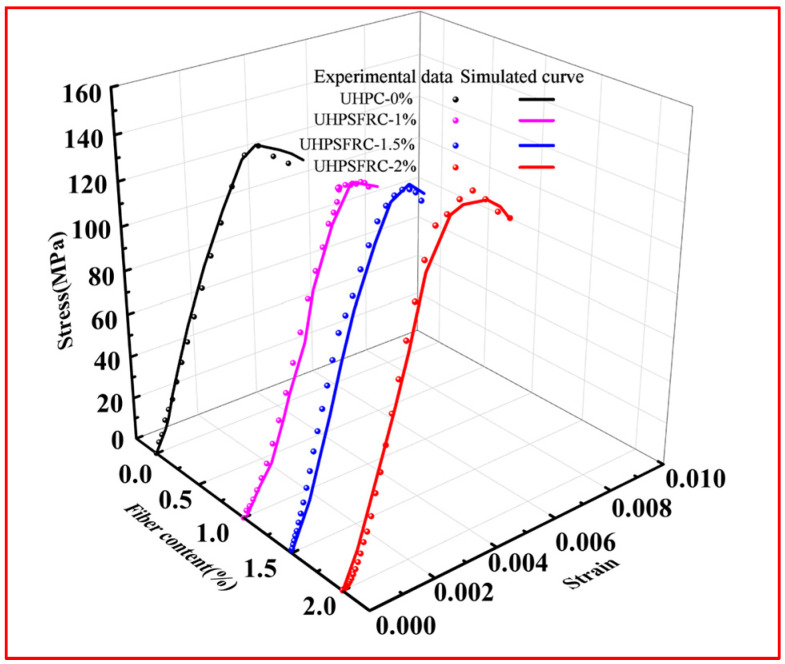
Comparison of experimental data and theoretical results at 200 s^−1^ strain rate.

The validity of the dynamic damage constitutive model was verified using experimental data at 200 s^−1^, as depicted in Figure 14. 

As can be seen from Figure 14, at a strain rate of 200 s^−1^, the theoretical results are in good agreement with the experimental data, which demonstrates that the dynamic damage constitutive model established in this paper, considering the influence of strain rate and fiber content, has a good applicability and can describe the mechanical behavior of UHPSFRC at a high strain rate.

## 5. Conclusions

(1) Under dynamic compression and quasi-static compression loads, there are obvious differences in the failure modes of ultra-high-performance concrete (UHPC) and ultra-high-performance fiber-reinforced concrete (UHPFRC). Under static loading conditions, the failure mode in the UHPC specimen is brittle shear failure, while the failure mode in the UHPSFRC specimen is tension failure. Under dynamic loading conditions, UHPC exhibits a brittle fracture failure mode, while UHPSFRC shows brittle–ductile failure characteristics of cracking but not crushing. The crack propagation inside the UHPC specimen is effectively inhibited by the steel fibers, and with an increase in fiber content, the toughness of UHPSFRC is gradually enhanced.
(2) The dynamic mechanical behavior of UHPSFRC is significantly affected by the strain rate strengthening effect. With an increase in strain rate, the dynamic peak stress of UHPSFRC significantly increases, while the peak strain slightly shrinks. The strain rate of 170 s^−1^ is a critical threshold at which the failure mode, dynamic compressive strength, and strain rate sensitivity of UHPSFRC are largely enhanced.
(3) The content of steel fiber has a significant effect on the dynamic mechanical properties of UHPSFRC. Specifically, when the fiber content is 1.5%, UHPSFRC exhibits lower crack propagation rates, superior impact cushioning properties, and a lower strain rate sensitivity. On the other hand, at a fiber content of 2%, UHPSFRC shows an optimal compressive strength.
(4) A new DIF-log$\dot{\varepsilon }$ model is proposed to describe the variation in strain rate sensitivity for UHPC and UHPSFRC. Based on the statistical damage theory and energy conversion principle, a dynamic damage constitutive model considering the influence of strain rate and fiber content is established, which can well describe the mechanical behavior of UHPSFRC at a high strain rate.

## Figures and Tables

**Table 1:** Raw material mix ratio of UHPSFRC (kg/m^3^).

Material	UHPC-0%	UHPSFRC-1%	UHPSFRC-1.5%	UHPSFRC-2%
Cement	870	870	870	870
Silica powder	307	307	307	307
Silica fume	260	260	260	260
Quartz powder	245	245	245	245
Fine aggregate	1055	1055	1055	1055
Superplasticizer	9	9	9	9
Water	180	180	180	180
Steel fiber	0	60	87	120

**Table 2:** Parameters for the new DIF model.

Parameter	UHPC-0%	UHPSFRC-1%	UHPSFRC-1.5%	UHPSFRC-2%
α	0.0719	0.07166	0.07146	0.07152
β	27	27	27	27
R^2^	0.981	0.978	0.954	0.988

**Table 3:** The fitting parameters of the proposed constitutive model.

$\dot{\varepsilon }$ (s^−1^)	Parameter	UHPC-0%	UHPSFRC-1%	UHPSFRC-1.5%	UHPSFRC-2%
90	$f(\dot{\varepsilon },\lambda )$	39.75	37.96	38	40.17
	a	0.0065	0.0067	0.0070	0.0066
	r	2.044	2.234	2.392	2.162
	u	−0.003	−0.003	−0.003	−0.003
	R^2^	0.976	0.992	0.977	0.957
120	$f(\dot{\varepsilon },\lambda )$	50	48.22	55.99	46.74
	a	0.0069	0.0071	0.0073	0.0070
	r	2.270	2.586	2.647	2.435
	u	−0.003	−0.003	−0.003	−0.003
	R^2^	0.986	0.991	0.992	0.972
140	$f(\dot{\varepsilon },\lambda )$	55	58.13	56.15	55.4
	a	0.0072	0.0076	0.0078	0.0074
	r	2.487	2.802	2.954	2.692
	u	−0.003	−0.003	−0.003	−0.003
	R^2^	0.961	0.996	0.997	0.998
170	$f(\dot{\varepsilon },\lambda )$	36	36.05	36.05	37.19
	a	0.0076	0.0079	0.0082	0.0078
	r	2.770	3.106	3.275	3.215
	u	−0.003	−0.003	−0.003	−0.003
	R^2^	0.996	0.992	0.993	0.993

## Data Availability

Data are contained within the article.
